# Intercomparison of radon and radon progeny concentration measurements performed in the Historic Silver Mine in Tarnowskie Góry, Poland

**DOI:** 10.3389/fpubh.2025.1681537

**Published:** 2025-10-15

**Authors:** Katerina Navratilova Rovenska, Miriam Slezakova, Caroline Vignaud, Pascale Blanchart, Katarzyna Wołoszczuk, Jostein Hoftuft, Agata Grygier, Krystian Skubacz, Arturo Vargas, Andrea Maiorana, Valeria Gruber, Joachim Gräser, Claudia Grossi, Victòria Moreno, Lluís Font, Karel Jilek

**Affiliations:** ^1^Statni ustav radiacni ochrany, v.v.i., Prague, Czechia; ^2^Direction de la recherche et de l'expertise en environnement, Autorité de Sûreté Nucléaire et de Radioprotection, Montrouge, France; ^3^Centralne Laboratorium Ochrony Radiologicznej, Warsaw, Poland; ^4^Direktoratet for strålevern og atomsikkerhet, Østerås, Norway; ^5^Główny Instytut Górnictwa-Państwowy Instytut Badawczy, Katowice, Poland; ^6^Institute of Energy Technologies, Universitat Politècnica de Catalunya, Barcelona, Spain; ^7^Centro Nazionale per la Protezione dalle Radiazioni e Fisica Computazionale, Istituto Superiore di Sanità, Rome, Italy; ^8^Österreichische Agentur für Gesundheit und Ernährungssicherheit (AGES), Abteilung Radon und Radioökologie, Linz, Austria; ^9^Physics Department, Universitat Politècnica de Catalunya, Barcelona, Spain; ^10^Departament de Física, Universitat Autònoma de Barcelona, Barcelona, Spain

**Keywords:** intercomparison, underground mine, radon concentration, radon progeny concentration, potential alpha energy concentration (PAEC), continuous monitor, integrative measurement

## Abstract

**Introduction:**

In the frame of the RadoNorm project, within work package 5.4, an intercomparison of radon and radon progeny measurements was organized in the Historic Silver Mine in Tarnowskie Gory (Poland). The aim of this intercomparison campaign was to compare the results of different electronic monitors for measurement of radon and radon progeny concentration under field conditions of an underground workplace over 3 days of measurements.

**Methods:**

In total, nine laboratories from seven European countries participated in the intercomparison study contributing with sixteen continuous radon monitors, ten radon progeny continuous monitors, and one TLD-based integrating system for PAEC measurements.

**Results:**

Despite the short duration of the field campaign, the comparison of radon activity concentration measurements showed strong consistency across most instruments, although notable deviations were observed with three instruments. Radon equivalent equilibrium concentration measurements also demonstrated good agreement, with only one outlier among ten instruments. Comparison of short term averages of EEC (PAEC) obtained from continuous monitors and integral TLD based Alpha probes showed good agreement. Greater variability was observed in the results for unattached radon progeny.

**Discussion:**

This intercomparison also allowed of testing instrument's responses in extreme ambient conditions with high humidity and relatively low temperature that are at the edge of the instrument's operating conditions.

## 1 Introduction

In Europe, the regulatory framework for radon and its progeny is primarily guided by the European Basic Safety Standards (EU BSS) Directive 2013/59/Euratom (1). This directive requires the Member States to establish a reference level for radon (^222^Rn) activity concentration in both new dwellings and workplaces, recommending a maximum level of 300 Bq/m3. Member States shall establish national action plans, which include strategies for radon measurement, mitigation, and public awareness. Overview of status of establishment and implementation radon action plans among EU Member States is presented in the report from EU-RAP study (2). Although there is only one Directive, radon action plans vary from one Member State to another, both in terms of strategy and implementation. Each individual country has considered national experience with radon issues, potential level of exposure of the population and other parameters. Requirements of the BSS on radon in workplaces are provided in the Publication RP-193 (3) and some details on measurement at workplaces is provided also in the Publication RP-188 (4).

Although measurement protocols established in Member States primarily focus on measurement of radon activity concentration (2), specific workplace conditions may require not only the measurement of radon gas, but also radon progeny. Following the European Commission's adoption (5) of all the dose coefficients published in ICRP's (International Commission of Radiological Protection) publications Occupational Intakes of Radionuclides: Part 1–5—including those for radon—the measurement of radon progeny has become even more important.

In order to apply a dosimetric model for calculating the effective dose due to the inhalation of radon progeny, the activity size distribution of the short-lived radon progeny, namely ^218^Po, ^214^Pb and ^214^Po (^214^Bi), should be measured. However, due to the difficulty in measuring these distributions, they are typically categorized into three size regions significantly affecting dose calculation: unattached, nucleation and accumulation (6). Several studies over the last decades have demonstrated the variability of aerosol conditions in different types of workplaces, as well as the variability of radon and radon progeny concentrations [summarized, for example, in ICRU Report 88 (8) and ICRP Publication 137 (6)]. Marsh and Birchall (49) performed a sensitivity analysis for residential environments to identify parameters that most strongly influence lung doses from inhaled radon progeny. Their main findings can be summarized as follows: changes in the unattached fraction significantly affect the lung dose, with higher unattached fractions increasing the dose due to greater deposition in the bronchial and bronchiolar regions; aerosol size of the unattached fraction is less influential than the nucleation fraction and nucleation aerosol size, but still the effective dose per unit exposure increases by almost 40 % as the activity median thermodynamic diameter (AMTD) of the unattached fraction increases from 0.5 nm to 3.5 nm; with the increasing nucleation fraction expressed in percentage of the total Potential Alpha Energy Concentration (PAEC), the weighted committed equivalent dose to the lungs per unit exposure to radon progeny increases; with the increasing aerosol size of the nucleation fraction weighted committed equivalent dose to the lungs per unit of exposure to radon progeny decreases due to the decreasing efficiency of diffusional deposition in the bronchial and bronchiolar regions; weighted committed equivalent dose to the lungs per unit exposure to radon progeny is quite insensitive to subject related parameters such as age, gender, but it is sensitive to target cell parameters such as depth, thickness of the target tissue; higher breathing rates increase the dose because more progeny are inhaled per unit exposure. Across the full range of parameter values, the weighted committed equivalent dose to the lungs per unit exposure varied between 8 mSv and 33 mSv per working level month (WLM).

Taking into account both dosimetry as well as epidemiology of radon progeny and associated exposure, the ICRP Publication 137 (6) recommends to use a single value of radon dose coefficient (RDC, Effective dose per unit of PAEC) of 3 mSv per mJ h m^−3^ and *F* = 0.4 where *F* is the equilibrium factor for calculation of doses following inhalation of radon and radon progeny in underground mines and in buildings. For substantial physical activity indoors, and for workers in tourist caves, the ICRP 137 Publication recommends using the RDC of 6 mSv per mJ h m^−3^.

When the equilibrium factor is measured and is found to be significantly lower or higher compared to the reference value of *F* = 0.4, it is more appropriate to determine F based on measurement and to determine also the unattached fraction *f*_*p*_ (4, 7).

Reliable data are essential for both routine radiation protection practice (3, 4, 6, 8) and scientific research. One of the tools for ensuring reliable data is comparison measurement. Comparison measurements are highly important for several reasons. The first reason is quality assurance. Regular comparison measurements help verify the accuracy, precision, and overall reliability of measurement instruments. If they are conducted on regular basis, systematic errors or calibration drifts can be identified and corrected, ensuring the instruments provide consistently accurate data. Another reason concerns the need to ensure consistency among different instruments, between manufacturers, in the case of highly specific equipment produced in small series or even for individual units, and laboratories. Furthermore, comparison measurements play an important role in meeting international guidelines such as SSG-32 of the IAEA (9) or WHO (10). Standards such as ISO 11665 (11), IEC 61577 (12) series or ISO/IEC 17025 (13) explicitly recommend or require regular participation in inter-comparison exercises.

Comparison measurements of continuous monitors in laboratory conditions in radon atmosphere or mixed fields of radon and thoron are performed relatively frequently as evidenced by the number of publications (14–21).

During the period of the laboratory inter-comparison, conditions are kept stable, and homogeneity of activity is ensured by various means (radon supply is controlled by certified radon/thoron sources with known exhalation rate and calibrated air flow meters, temperature, relative humidity, and air exchange rate is controlled by ventilation system, particle size distribution and concentration is controlled by aerosol generator, homogeneity of radon and thoron gas concentration in the measured volume can be controlled by fans and grab sampling). In contrast to that, at typical workplaces, including underground workplaces, ambient conditions and radon/radon progeny concentrations are variable in time. Sometimes ambient conditions or concentration of radon/radon progeny appear to be on the far edges of operating ranges of continuous monitors.

Although comparison measurements in field conditions may initially be perceived as redundant or difficult to evaluate, their implementation proves to be highly beneficial for both applied research and operational practice because they provide information about the behavior of instruments in the conditions in which they are actually used. Changes in conditions potentially affecting the detector response may have effects that amplify or cancel each other out.

Unfortunately, reports on comparison measurement carried out under field conditions are much less frequent compared to those carried out under laboratory conditions (22–24).

In the RadoNorm project “Toward effective radiation protection based on improved scientific evidence and social considerations—focus on Radon and NORM”, work package 5.4., underground workplaces were studied. As part of this work intercomparison studies of the activity concentration of radon and radon progeny were conducted in the conditions of an underground workplace. These studies were held in the Historic Silver Mine in Tarnowskie Góry (Poland) from 20 to 23 February 2024.

The main goal of these measurements was to compare the results of currently available continuous monitors for radon and radon progeny in real conditions of an underground workplace.

## 2 Materials and methods

### 2.1 Participants

A total of nine laboratories from seven different countries participated in these studies, namely French Authority for Nuclear Safety and Radiation Protection (ASNR, France), Central Laboratory of Radiological Protection (CLOR, Poland), Norwegian Radiation and Nuclear Safety Authority (DSA, Norway), Central Mining Institute—National Research Institute (GIG, Poland), Italian National Institute of Health (ISS, Italia), Austrian Agency for Health and Food Safety (AGES, Austria), National Radiation Protection Institute (SURO, Czech Republic), Autonomous University of Barcelona (UAB, Spain) and Polytechnic University of Catalonia Universitat Politécnica de Catalunya (UPC, Spain). Each laboratory received a unique ID code, which is provided alongside its results.

### 2.2 Characteristics of the measurement site

**Figure 1:**
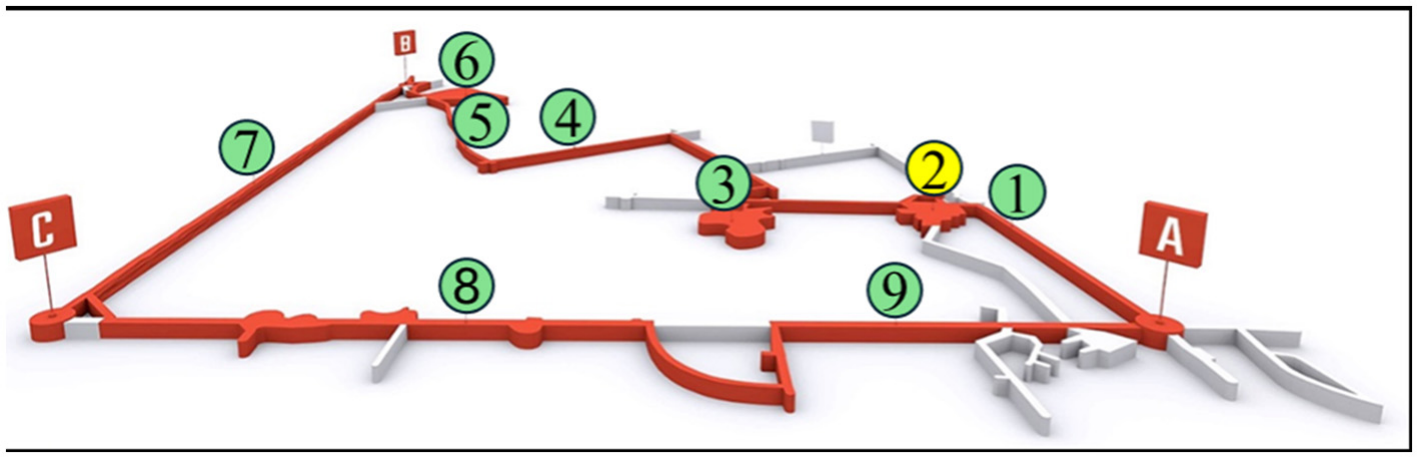
Tourist route in the Historic Silver Mine (A—“Anioł” shaft: B—“Szcześć Boże” shaft; C—”Żmija” shaft; 1—“Staszic” gallery; 2—“Srebrna” chamber; 3—”Zawałowa” chamber; 4—“Niski” gallery; 5—”Wysoki” gallery; 6—“Niska” chamber; 7—water gallery (boat flow); 8—”Okreżny” gallery; 9—“Garus” gallery) (29).

The Historic Silver Mine is located within the city limits of Tarnowskie Góry. Exploitation of deposits in the area was completed in 1912, and since 1976 some of the former excavations have been opened to the public. During the operation of the Frederick (former name) mine, 150 km of workings and about 20,000 shafts and shafts were created since 1,784 (25, 26). Most of the former mine excavations have been isolated and the remainder has been used for tourism facilities. The current route open to the public includes galleries 1,740 meters long in the area of the 3 mine shafts Anioł, Żmija and Szcześć Boże, with the Szcześć Boże shaft buried and having no technical function. The mine is located at a depth of 40 m. The tour time of the mine is about 1 h. A diagram of the tourist route is shown in Figure 1. The accessible part of the mine has a specific microclimate, characterized by a fairly constant temperature of about 10 °C throughout the year and relative humidity of about 90 %.

The ore minerals in the area where the Historic Silver Mine in Tarnowskie Góry is located occurred in carbonate Triassic sediments, which are part of the Permian-Mesozoic platform cover. The Triassic in the Upper Silesian region is fully developed. The crumbling of the deposit is associated with a series of so-called crushed-bearing dolomites, characterized by vertical and horizontal variation in formation. The excavations currently included in the underground tourist route were made in these rocks. Stratigraphically, they correspond to the Early and Middle Triassic, and their age is 235–252 million years (27). Structurally, they are crystalline to compact rocks, generally cavernous and fractured, mostly coarse to medium-bedded, interlayered with layers of weakly compacted dolomite or clay. In the area of the mine, they lie relatively shallow, 20–50 m from the surface. As a result of tectonic phenomena, the complex has been severely cracked (a dense network of cracks and fissures). With tectonic phenomena are associated with later karst processes. The radon risk present in the Historic Silver Mine is associated with these very described phenomena. Furthermore, radon from workings that are not part of the tourist route can infiltrate into the accessible part of the mine through leaky dams. The main ore mineral in the area in question was galena (PbS), called silver-bearing galena because of its high silver content (up to 1.2 % maximum). The galena deposits were located mostly in the bottom part of the ore-bearing dolomites, and the ore occurred in the form of veins and vein-like shoals. In addition, zinc minerals in the form of sphalerite and wurtzite were sporadically present in the deposit (28).

The measurements of activity concentrations of natural nuclides in rock and water samples were made by the Central Mining Institute (Katowice, Poland) using gamma spectroscopy (Canberra Packard Central Europe, Schwadorf, Austria) for rock samples and liquid scintillation spectrometry (Quantulus, PerkinElmer, Shelton, United States) for water samples. Maximum measured activity concentrations in rock samples were 19 ± 1 Bq/kg (^226^Ra), 5 ± 1 Bq/kg (^228^Ra), and 5 ± 1 Bq/kg (^228^Th) while the activity concentration in waters did not exceed 0.01 kBq/m^3^ for ^226^Ra and was < 0.06 kBq/m^3^ for ^228^Ra (48).

Ventilation of the mine is carried out in two ways. When it is not open to tourist activity, the route is ventilated by means of natural depression in the direction from the “Żmija” shaft to the “Anioł” shaft. When it is open to tourist use, the underground route is ventilated mechanically. A suction fan is installed in the “Anioł” shaft to exhaust the air. This fan ensures air flow in the excavations of the underground route in the amount of at least 120 m^3^/min. In order to ensure rapid and effective air exchange in the excavations, the underground route is additionally ventilated for approximately 1.5 h after the start of tourist activity. This is achieved by operating one of the two fans installed at the ventilation station at the ‘Żmija' shaft, generating positive-pressure ventilation in the direction from the ‘Żmija' shaft to the ‘Anioł' shaft. The ventilation system is not equipped with filters; it simply moves the existing air through the excavations.

The measurement campaign was carried out in the “Srebrna” (Silver) chamber at a depth of 40 m (Figure 1). The dimensions of the chamber are as follows: length 25 m, width 25 m and height 3 m. According to the mine ventilation specialists, mechanical ventilation was in operation on each measurement day from 08:40 to 15:00. However, the “Srebrna” chamber is located next to the main air stream, so ventilation in this area is much less effective compared to the main mine corridors.

A series of measurements of ventilation network parameters were taken at the Historic Silver Mine to create a model and implement it into the Ventgraph software (30). The air exchange in the chambers was very poor. However, there where flow rate measurements were possible, significant disparities were observed between the flow rates in the chambers and in the adjacent gallery, reaching as high as 1:35. The airflow rate in the gallery running next to the “Srebrna” chamber was 33 m3/min, while in the chamber itself it was impossible to determine this parameter and it must have been < 1 m3/min which was the limit of flow rate measurement for the applied devices. For the flow velocity the limit was 0.15 m/s.

### 2.3 Instrumentation and quantities of comparison

**Figure 2:**
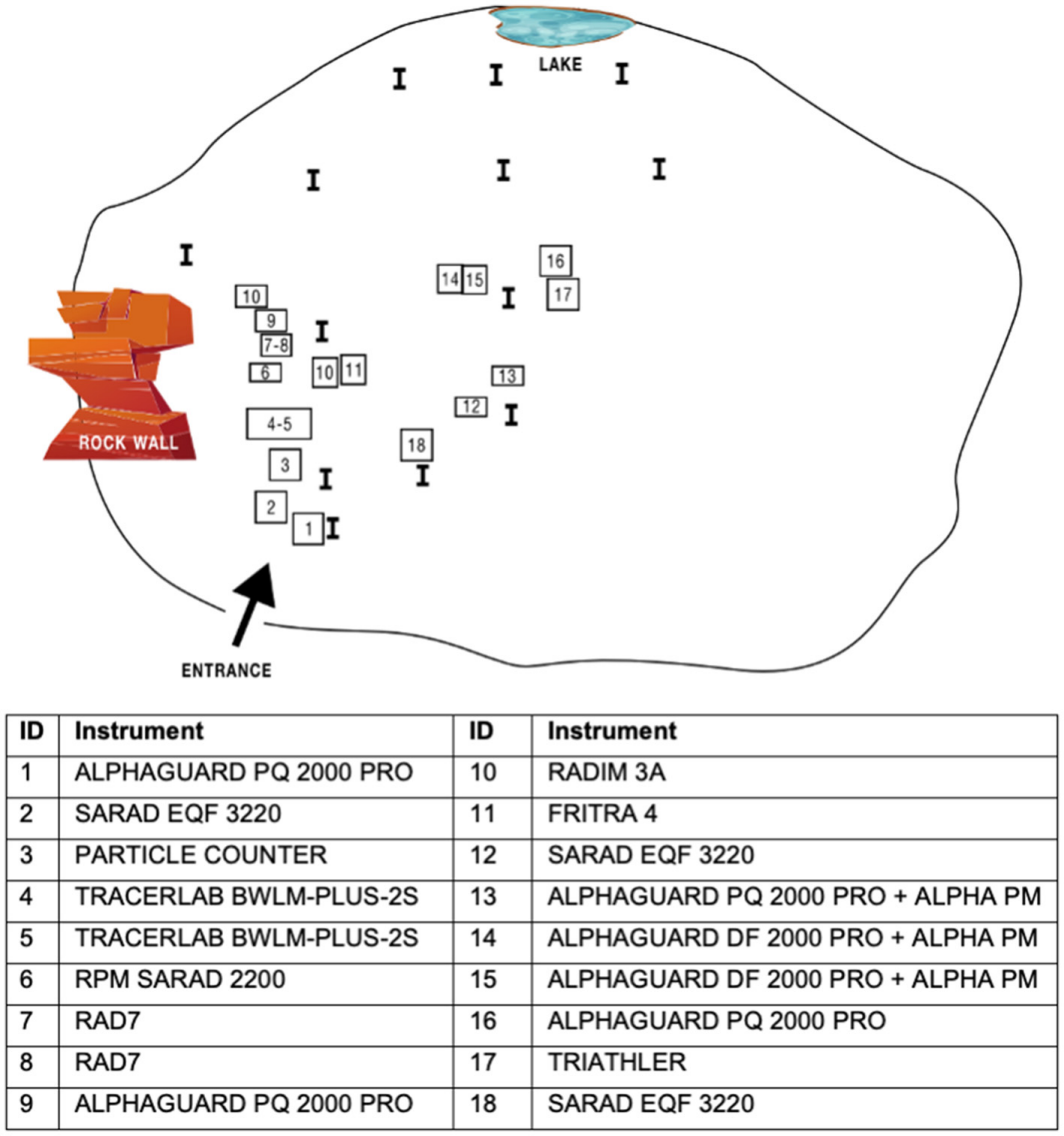
Distribution of measurement technique across the “Srebrna” chamber.

In comparative measurements, devices designed to measure radon concentration (in total 16 devices, Rn) and the concentration of radon progeny (in total 10 devices, PAEC—potential alpha energy concentration—and EEC—equilibrium equivalent concentration) were used. Some of them were also equipped with modules for determining the share of the unattached fraction (in total 6 out of 10). One participant provided an integral system based on TLD for measurement of PAEC. Indication of distribution of devices across the “Srebrna” chamber is depicted on Figure 2; the real situation is depicted on Supplementary Figure 1. The following instruments took part in the inter-comparison:

- AlphaGUARD by Bertin (formerly Genitron-Saphymo) is a high-precision radon monitor using a pulse ionizing chamber for continuous radon measurement in air. It operates in both diffusion and flow-through modes and records radon activity concentration, temperature, pressure, and humidity. For this comparison measurement, diffusion mode was used (31).
- AlphaGUARD+AlphaPM—AlphaPM is an add-on module for AlphaGUARD that measures airborne radon progeny concentrations in parallel to AlphaGUARD's measurement. AlphaPM samples air and collects aerosol with radon progeny on a filter. Semiconductor detector PIPS is used to count the alpha activity. Flow-rate is set to 2 l/min (32).
- RAD 7 by Durridge (USA) is an electronic radon detector using an alpha spectroscopy with a solid-state silicon detector to provide radon and thoron concentration readings. Depending on the setting, it may have rapid response in sniffing mode. Drying tube or Drystik is necessary for correct performance of this detector. Flow-through mode is only available (33).
- RADIM3a by SMM-Jiri Plch (Czech Republic, not on the market anymore) measures radon concentration via alpha detection of ^218^Po collected electrostatically. It includes sensors for temperature, humidity, and pressure, with results corrected for environmental conditions. Diffusion mode is only available.
- BWLM-PLUS-2S by Tracerlab (Germany, not on the market anymore) is a 2-channel radon and thoron progeny monitor with two independent samplers. One with filter for total progeny activity concentration measurement, second is with mesh for unattached fraction measurement. Alpha-spectrometry is done by silicon detector. BWLM-PLUS-2S has three different measurement modes (Markov, Nuclide and Continuous). Two of them were used during this inter-comparison: Nuclide mode, where EEC and activity concentration of single nuclides (218Po, 214Pb, 214Bi/Po) are given after 1h cycle; and Continuous mode, based on continuous sampling for EEC determination in preset counting interval and providing results from two calculation methods; slow and fast (34).
- FRITRA 4 by SMM-Jiri Plch (Czech Republic, not on the market anymore) is a continuous monitor for radon progeny (EEC, equilibrium equivalent activity concentration) using alpha spectroscopy and fixed 2 h sampling interval. Main advantage is the open face mesh-filter sampling head posing no obstacles for aerosol along the path from measured atmosphere to the measuring head.
- EQF 3220 by Sarad (Germany) measures simultaneously radon, thoron, and progeny, including particle-size-specific aerosol fractions—unattached fraction, nucleation fraction in the range of 10–100 nm, and attached fraction. Alpha-spectroscopy is carried out by 4 silicon detectors with hi-voltage chambers in fast (excl. ^214^Po) and slow (incl. ^214^Po) mode; radon progeny are sampled via telescopic sampling head equipped with 2 ion-implanted silicon detectors. Flow-rate is set to 1.65 l/min and 1.5 l/min. Results of radon activity concentration are available from two modes—fast mode, which includes only ^218^Po, and slow mode, which includes both ^218^Po and ^214^Po decay (35).
- RPM 2200 by Sarad (Germany) is radon progeny monitor. Radon progeny deposited on ambient aerosol are sampled and collected on a fine pore membrane filter. Measurement of the potential alpha energy concentration (PAEC) is done by silicon detector. Flow-rate is set to 1.5 l/min (36). PAEC can be converted to EEC by dividing it by a factor 5.57 × 10^−9^ J Bq^−1^ (8).
- The Alpha probe is designed to measure the average potential alpha energy concentration of radon and thoron progeny, designed and produced at GIG, Poland (37). The essential elements of the probe are the thermoluminescent CaSO_4_:Dy detectors put in three measuring heads located above the filter, on which the aerosols are collected. Each of them is equipped with two detectors, one of which, located directly above the filter, records also alpha radiation. This radiation does not reach the second detector, separated by a spacer and is designed to measure the radiation background. The use of three detectors decreases the lower limit of detection and allows for taking into account the uneven distribution of aerosols on the filter. The alpha meter works with an aspirator, which ensures a stable air flow through the filter and 8-h autonomous operation. The device is intrinsically safe and can be used in difficult environmental conditions. The lower limit of detection is in the order of 0.1 μJ/m^3^.

Finally, we also monitored the concentration of particles using the TSI Condensation Particle Counter (CPC) Model 3007, a portable instrument designed for the detection and counting of airborne ultrafine particles. It uses the principle of condensation particle counting to detect particles from 10 to 1000 nm in diameter and can measure concentrations up to 100,000 particles/cm3 with one-second resolution (38).

Most of the monitors for radon activity concentration had recent calibration done in calibration facility. On the other hand, many of monitors for radon progeny measurement did not have any recent calibration except of the manufacturer calibration. It should be noted that there is currently no primary standard for the radon progeny (total EEC) and no corresponding metrological traceability around the Globe for the unattached EEC. However, calibration of the total EEC is provided by several accredited laboratories.

The devices were distributed over an area of about 50 m^2^, with their inlets positioned at heights ranging from 30 to 60 cm.

### 2.4 Data processing

To conduct interlaboratory comparisons, the homogeneity and stability of measured quantities must be assured. This is not possible in the field conditions, where radon and radon progeny concentrations are naturally changing over time due to variability of the environment. Since the concentrations depend on both the date and time of day, time-series analysis is more appropriate in this situation than standard mean comparisons. Statistical methods provided by ISO 13528 do not consider analysis of time series data.

The instruments collected data in different sampling intervals-−10 min, 30 min, 1 h or 2 h (see Table 1), the time synchronization was not carried out at the beginning of the comparison measurement. For technical reasons, it was not possible to start the measurements simultaneously for all monitors. In addition, some instruments experienced technical issues and had to be stopped and restarted after the problems were resolved. The time settings of the monitors were checked at the beginning. To make the results comparable and the graphical representations clearer, the individual measurements from instruments with shorter sampling intervals were averaged to be on 2-h basis. The averages were calculated from the results with actual times closest to the considered 2-h intervals.

**Table 1:** List of registered instruments, corresponding measured quantities.

**Instrument type**	**Measured quantity**	**Number of instruments**	**Sampling interval used**
AlphaGUARD	Rn	3	10, 60 min
RAD 7	Rn	3	30, 60, 120 min
Radim3a	Rn	3	30 min
BWLM-PLUS-2S	Rn, EEC, EEC_U_	2	10, 30, 60 min
FRITRA 4	Rn, EEC, EEC_U_	1	120 min
EQF 3220	Rn, EEC, EEC_U_	3	10, 60 min
RPM 2200	PAEC	1	60 min
AlphaGUARD+AlphaPM	Rn, EEC	3	10, 60 min
α- probe (TLD)	EEC	2	integrative

Rn, radon activity concentration; EEC, equilibrium equivalent concentration; EEC_U_, equilibrium equivalent concentration unattached; PAEC, potential alpha energy concentration, number of instruments per type and sampling interval.

Only few stationary intervals lasting 6–8 h, where the concentrations showed approximately stable behavior, were identified in measured data. The stationary period was defined as the time interval during which the Rn (EEC) values remained approximately constant, with no apparent trend. The start of each stationary period was determined using the following method: 1-h, 2-h, … up to 8-h moving averages were applied to all measured data. The time points at which the range among these eight smoothed curves was minimal (i.e., the 1-h, 2-h, … and 8-h averages closely agreed) were considered the starting points of the stationary periods. Four such local minima were identified. In these time periods, methods in accordance with ISO 13528 (39) were applied. For each stationary period, averages and standard deviations were calculated. The results from the stationary periods were then analyzed using meta-analysis methods (40). A random-effects model was applied to the mean values and their associated uncertainties. This model assumes that variability among instruments arises not only from measurement errors but also from systematic (non-random) errors, for example due to poor calibration. Variability arising from sources other than measurement errors is referred to as heterogeneity. The grand mean—an estimate of the true quantity—is calculated as the weighted average of the mean values from all instruments, with weights based on their measurement error variances and the heterogeneity estimated by the model.

The considered random-effects model is:


$$\begin{array}{l} y_{i}=m+M_{i}+e_{i},\;i=1,\ldots ,k\; \end{array}$$

where *y*_*i*_ is the observed value of the *i*-th instrument, *m* is the true value of the quantity, *M*_*i*_ is a random effect representing the bias of the i-th instrument from the true value and *e*_*i*_ is the measurement error of the i-th instrument. It is assumed that the random effect is normally distributed $M_{i}\;\sim N\left(0,\;\tau^{2}\right)$, where τ^2^ denotes the amount of heterogeneity among the instruments. The random errors are normally distributed $e_{i}\;\sim \;N\left(0,\;s_{i}^{2}\right)$, where the standard deviations *s*_*i*_ are known (calculated uncertainties). The goal is to estimate the true quantity *m* and the heterogeneity τ^2^.

The true value *m* was estimated as a weighted average of measurements with weights equal to $w_{i}\;=\;1/\left(s_{i}^{2}\;+{\hat{\tau }}^{2}\right)$, where ${\hat{\tau }}^{2}$ is the estimate of the heterogeneity from the model (restricted maximum-likelihood estimator). The homogeneity of the instruments was tested by Cochran's Q test ($H_{0}:\tau^{2}\;=\;0$). A *p*-value of the test less than the level 0.05 suggests that the heterogeneity is significant. However, this test has some limitations. It is only an approximative test and in case of few studies (here instruments) it has a low power. Therefore, a non-significant result must not be taken as evidence of no heterogeneity.

Another heterogeneity measure is the *I*^2^ index. It provides an estimate of the percentage of the variability that can be attributed to the heterogeneity of the instruments rather than to measurement errors. It is based on the Q value from the Cochran's test. A value of < 25 % is usually viewed as low heterogeneity, a value between 25 % and 50 % as moderate and a value larger than 50 % as high heterogeneity.

Uncertainties were provided by the participants according to their respective methodologies for assessing measurement uncertainty. Further discussion with participants revealed that the contents of the uncertainty budget vary, making the uncertainties incomparable, therefore only the statistical error was considered in the evaluation. The uncertainty of the mean was calculated as the standard error of the mean, i.e., standard deviation of the measurement values divided by the square root of the number of measurements in the interval.

The results are presented in the form of forest plots and include the grand mean for each investigated campaign. The data analysis was done in the R software environment (41).

## 3 Results

This chapter provides summary of obtained results. Sub-chapters are organized according to the measured quantities.

### 3.1 Radon activity concentration

Radon activity concentration measurement results were submitted and then evaluated for 15 continuous instruments of different types, some of the monitors did not provide full data sets. Sampling intervals were different, see Table 1, therefore 2-h averages were calculated for instruments with more frequent sampling. From the 2 hourly average time series it can be seen, that in the second half of the studied period the RADIM instruments start to diverge. The hypothesis to explain this effect is that the filter material protecting the detection chamber absorbs moisture, which in turn reduces the detection efficiency, among other possible influences.

From the time series of 2-h averages with no missing values an average curve was calculated (12 instruments out of 15 monitors were included).

**Figure 3:**
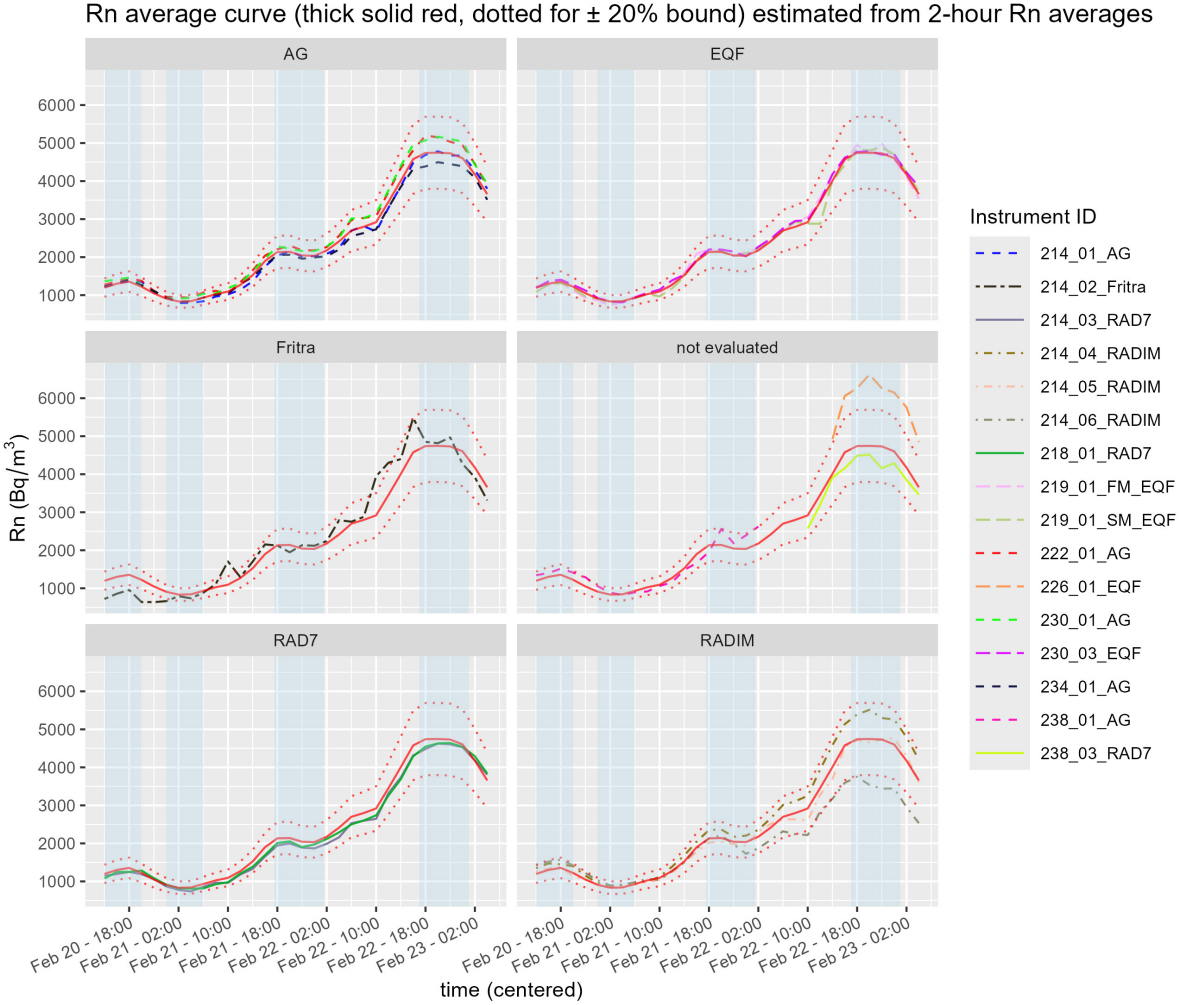
Time course of radon concentration (Rn) as measured by different instruments during the comparison measurement. Stationary periods are marked as a blue band.

The time course of radon activity concentration is presented in Figure 3. The average trend is shown as a thick red solid line, with ±20 % confidence bounds indicated by dotted lines. The instruments are grouped together according to their type. The instruments not used for the calculation of the average curve, i.e. with some missing data, are in the group denoted as “not evaluated”.

From the three-day time series four stationary periods were selected, where the concentrations were approximately stable and no trend was evident. The results from the stationary time periods were analyzed by methods of meta-analysis (see Section 2.4).

Identified periods were:

- P1 (6 h long): 20.2. 14:00−20.2. 20:00
- P2 (6 h long): 21.2. 0:00−21.2. 6:00
- P3 (8 h long): 21.2. 17:30−22.2. 1:30
- P4 (8 h long): 22.2. 17:00−23.2. 1:00

Since the approaches to assessing the uncertainty of the calculated mean values reported by the participants were not comparable, only the statistical error was considered in the evaluation. The uncertainty of the mean was calculated as the standard error of the mean, that means, standard deviation of the measurement values divided by the square root of the number of measurements in the interval.

Forest plot shows the measured mean values of the instruments together with their confidence intervals (using standard error of the mean), their contribution to the grand mean (weight in %) and the grand mean estimated as a weighted average of the instrument means. The red dotted lines are ±5 % and ±20 % deviations from the grand mean. Below the graph, the value of the statistic *I*^2^ together with its confidence interval is shown, square root of the estimate of the heterogeneity τ and the p-value of Cochran's homogeneity test (if *p* < 0.05, homogeneity is rejected).

**Figure 4:**
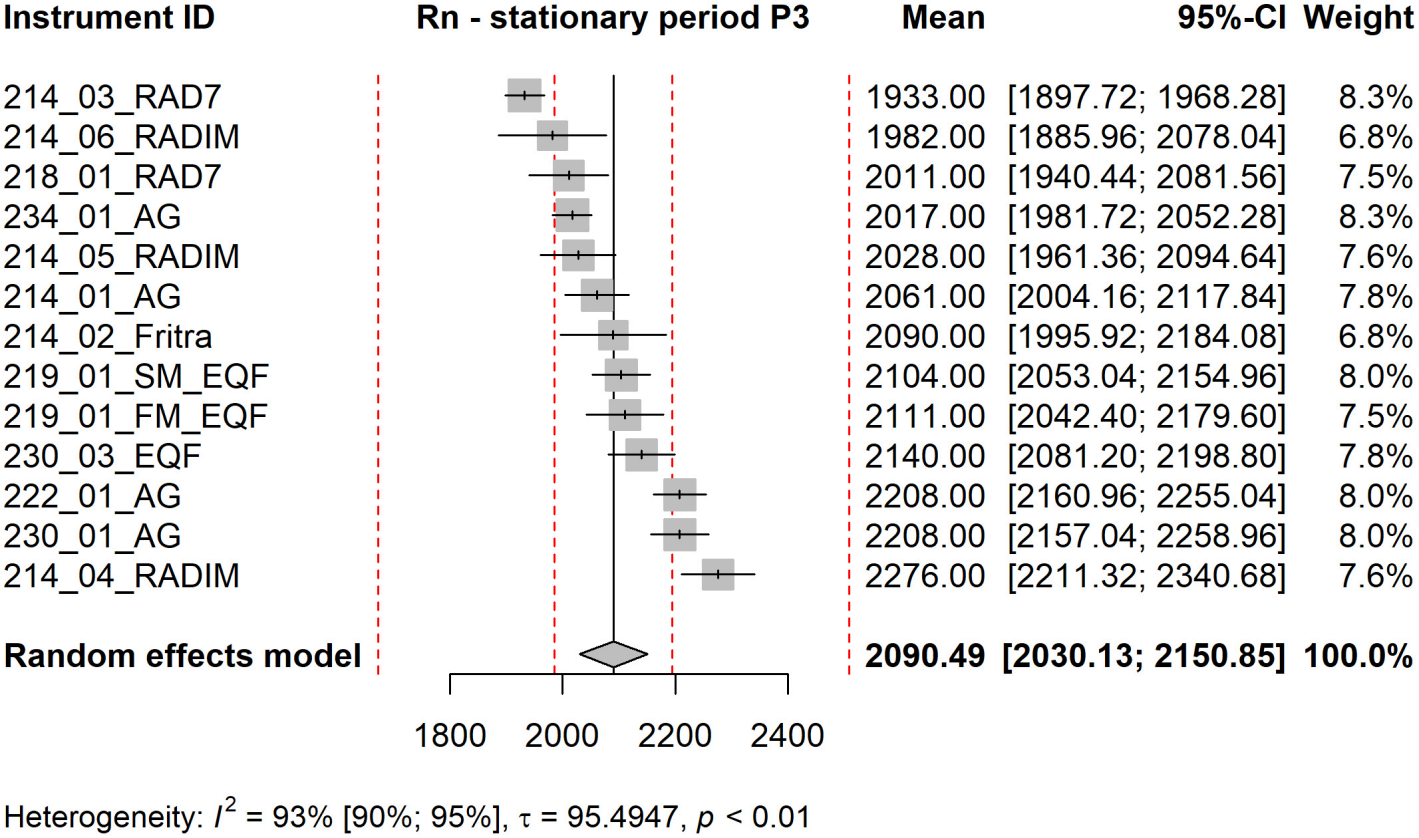
Forest plot for stationary period P3, Rn measurement. The red dotted lines are ±5 % and ±20 % deviations from the grand mean.

**Figure 5:**
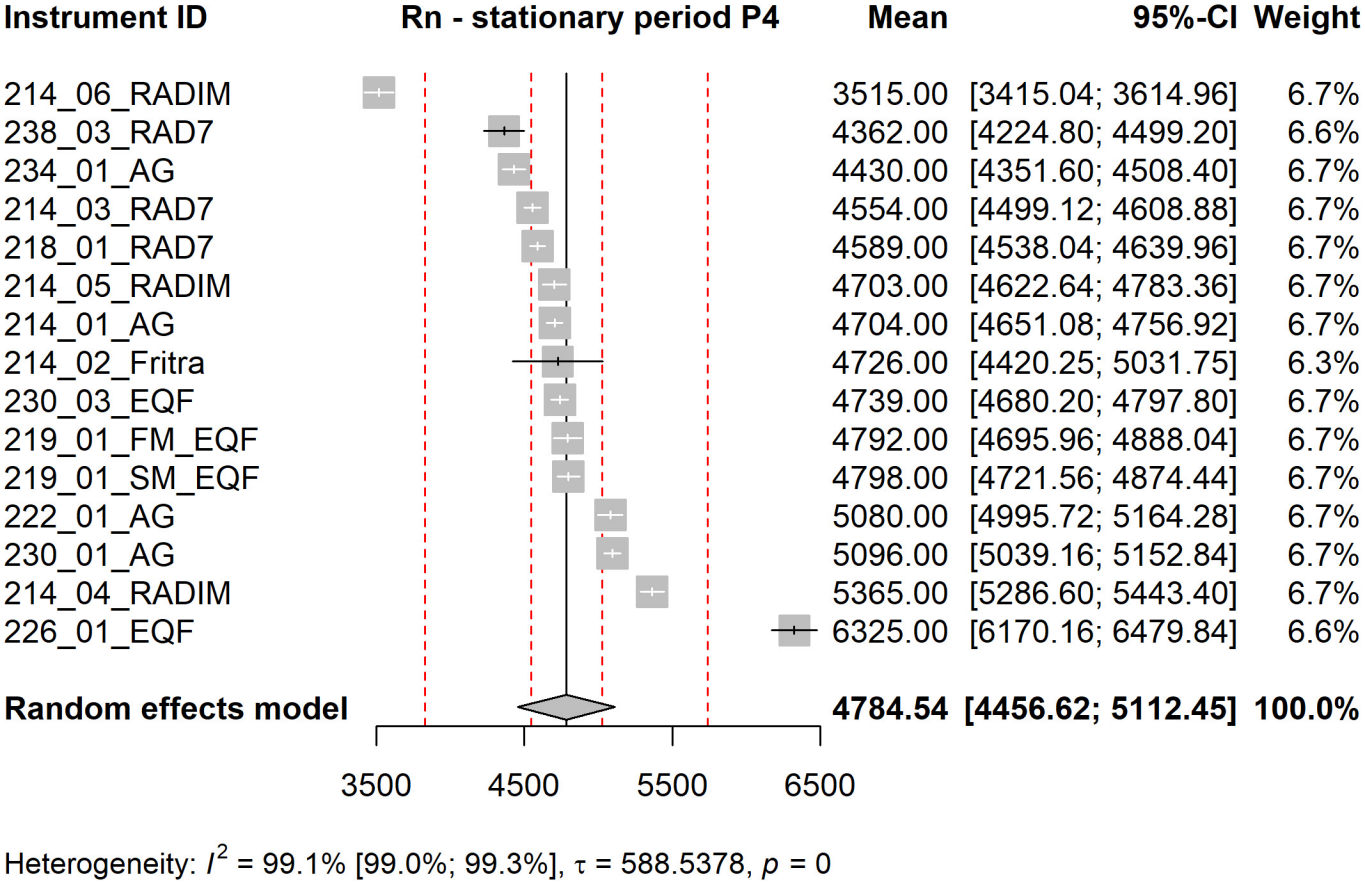
Forest plot for stationary period P4, Rn measurement. The red dotted lines are ±5 % and ±20 % deviations from the grand mean.

The forest plots corresponding to stationary periods P1 and P2 are included in the Supplementary Material as Supplementary Figures 2, 3. Forest plots for P3 (see Figure 4) and P4 (see Figure 5) are presented in the main text of this manuscript, along with corresponding EEC measurement results discussed in the next section. From Figures 4, 5 it is evident that the standard errors of the means are relatively small. More than 90 % of the variability is attributable to heterogeneity. This high level of heterogeneity arises because only the standard errors of the means are considered in the evaluation. All results from continuous radon monitors in the P1–P3 fall within the ±20 % of the grand mean. One of the RADIM3A monitor and one EQF3220 are outside this band in P4.

### 3.2 Equilibrium equivalent concentration (EEC)

Results for EEC measurement were provided by 9 instruments-−2 AlphaPM, 3 EQF 3220, 2 BWLM-PLUS-2S, 1 FRITRA, 1 RPM2200. The saved times of the instruments were centered in the middle of the 2 h intervals.

Participants with BWLM-PLUS-2S instruments decided to change instrument modes during their measurement (nuclide, continuous slow and continuous fast), therefore there is no continuous time series for the whole time period from BWLM-PLUS-2S monitor. On the other hand, the changes were carried out jointly, so the BWLM-PLUS-2S can be at least compared one to another. The 219-EQF 3220 had a drop out of 1 h and half (same as for Rn measurements). The 226-EQF 3220 started the third day of the exercise due to prolonged stay at customs.

**Figure 6:**
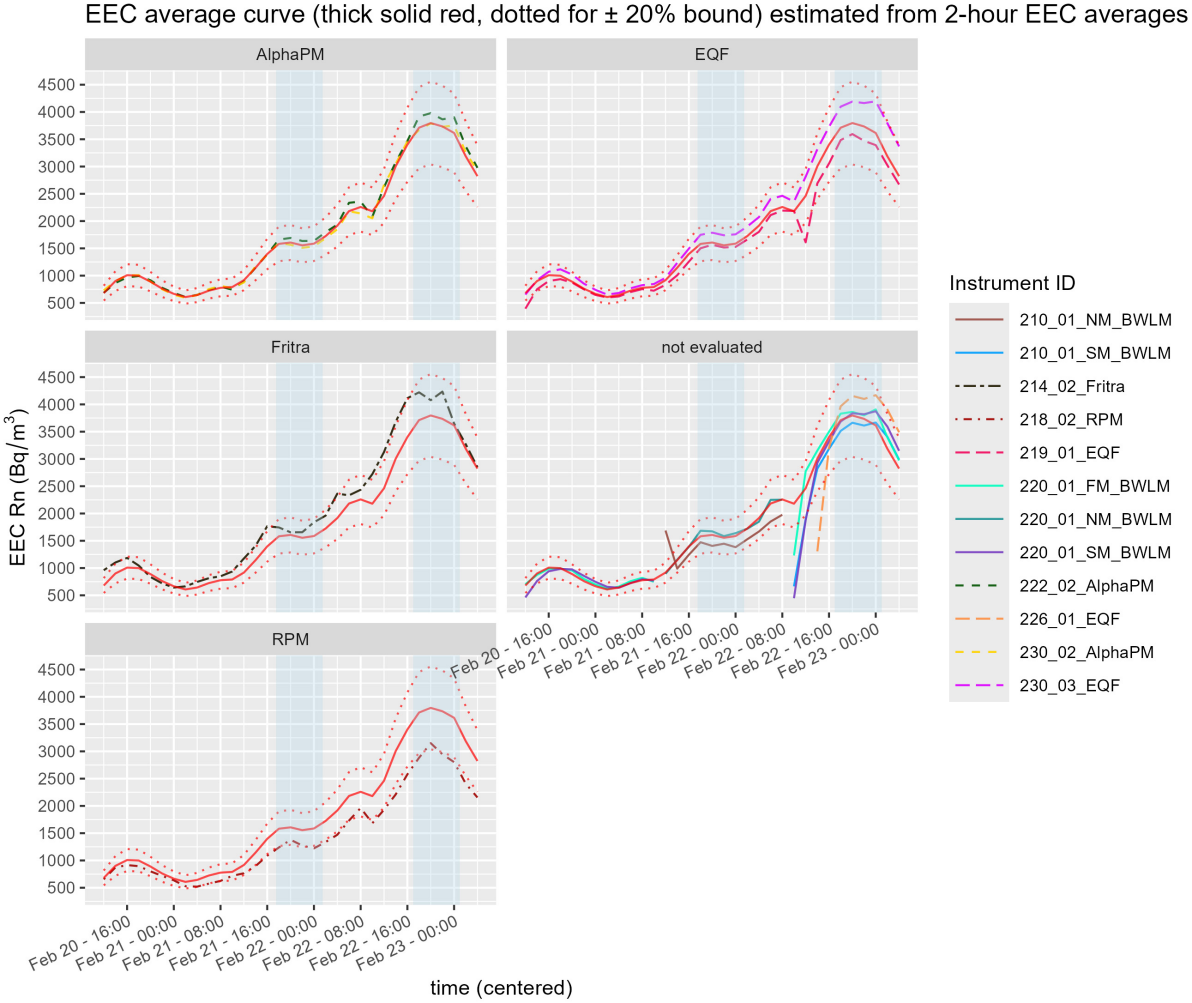
Time course of equilibrium equivalent activity concentration as measured by different instruments during the comparison measurement in the “Srebrna” chamber. Stationary periods are marked as a blue band.

The time course of equilibrium equivalent concentration is presented in Figure 6 which also shows the average curve—calculated using only the complete datasets—as a thick red solid line with ±20 % bounds indicated by dotted lines. The instruments not used for the calculation of the average curve are included in the graph and denoted as “not evaluated”. Both the BWLM-PLUS-2S (especially in slow mode) and EQF 3220 had slow start-up which can be seen very clearly in the graph. Almost all the curves lie within the ±20 % bound from the average curve. The exception is the RPM instrument, following approximately the lower 20 % bound.

From the three-day time series two stationary and sufficiently long periods were selected, where the concentrations were approximately stable and no trend was evident. These periods match with P3 and P4 period from radon gas measurement and are 8 h long. For each stationary period and each instrument mean EEC concentrations were calculated. The results from the stationary time periods were analyzed by methods of meta-analysis.

**Figure 7:**
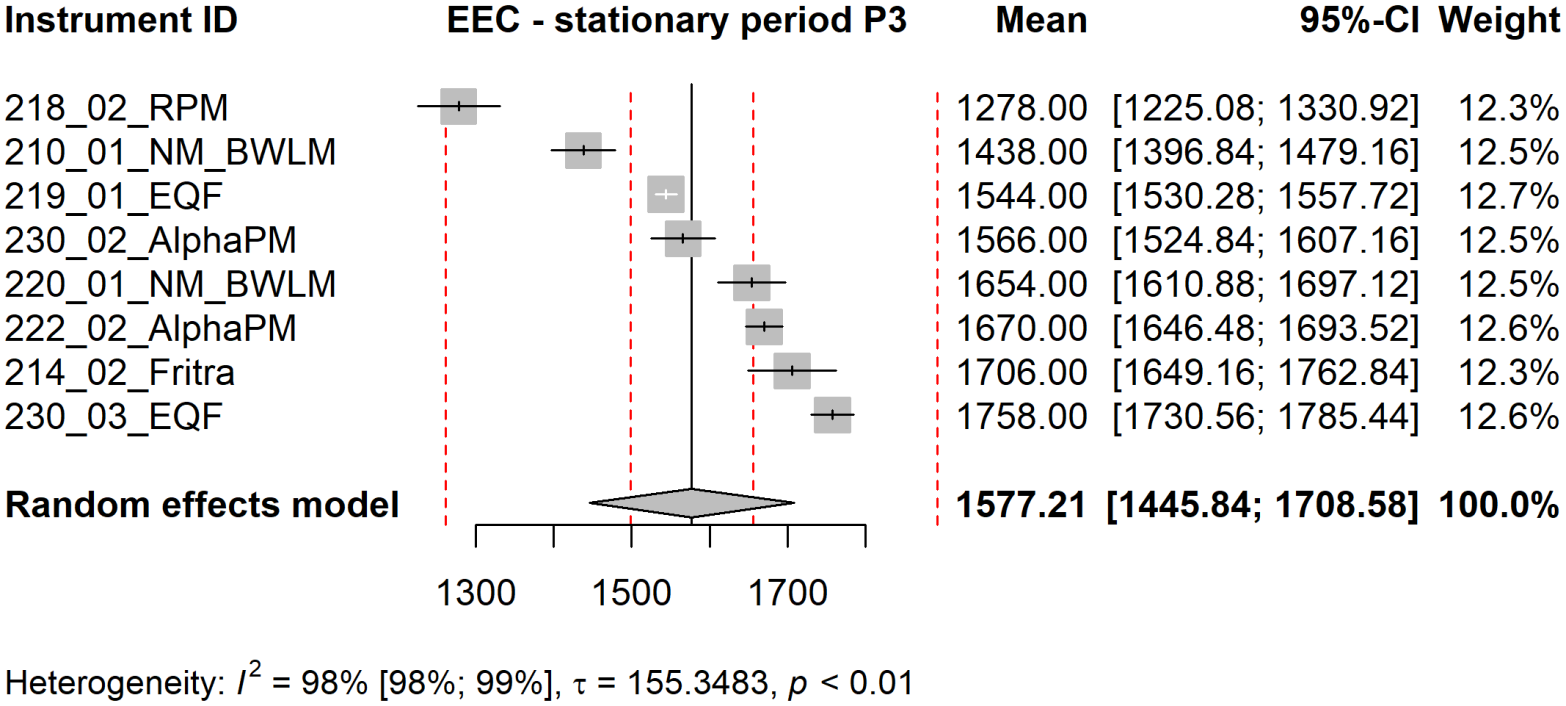
Forest plot of stationary period P3, EEC measurement. The red dotted lines are ±5 % and ±20 % deviations from the grand mean.

**Figure 8:**
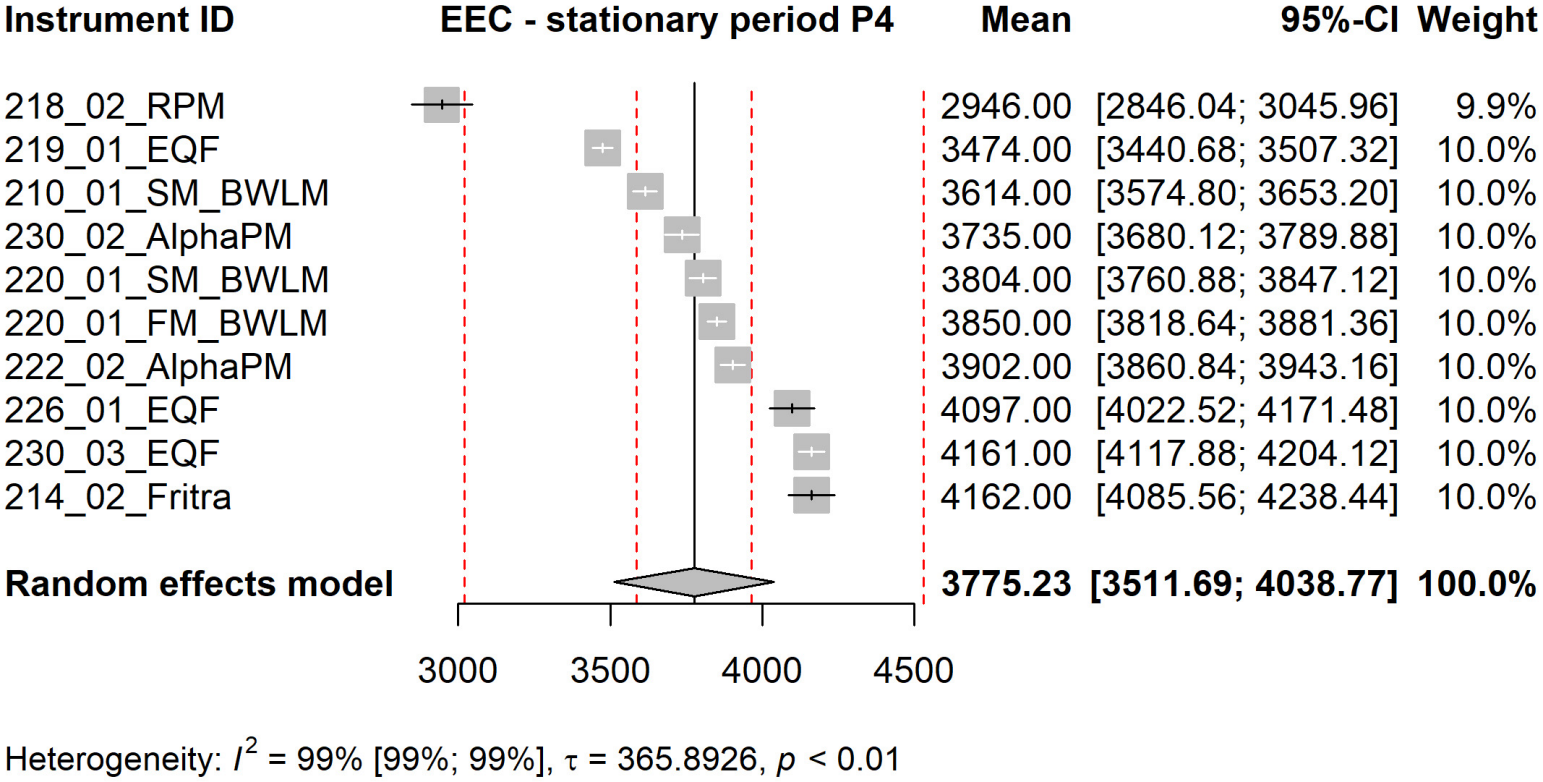
Forest plot of stationary period P4, EEC measurement. The red dotted lines are ±5 % and ±20 % deviations from the grand mean.

For period P3 (see Figure 7), the mean value of the RPM instrument is at lower 20 % boundary of the grand mean. For P4 (see Figure 8), it falls outside this boundary. Without the RPM, the grand mean would shift slightly to the right.

**Figure 9:**
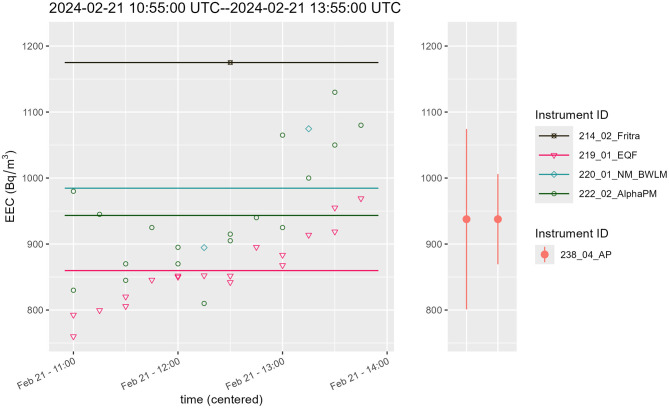
Comparison of EEC continuous **(left)** and integral measurement **(right)**, period II.

**Figure 10:**
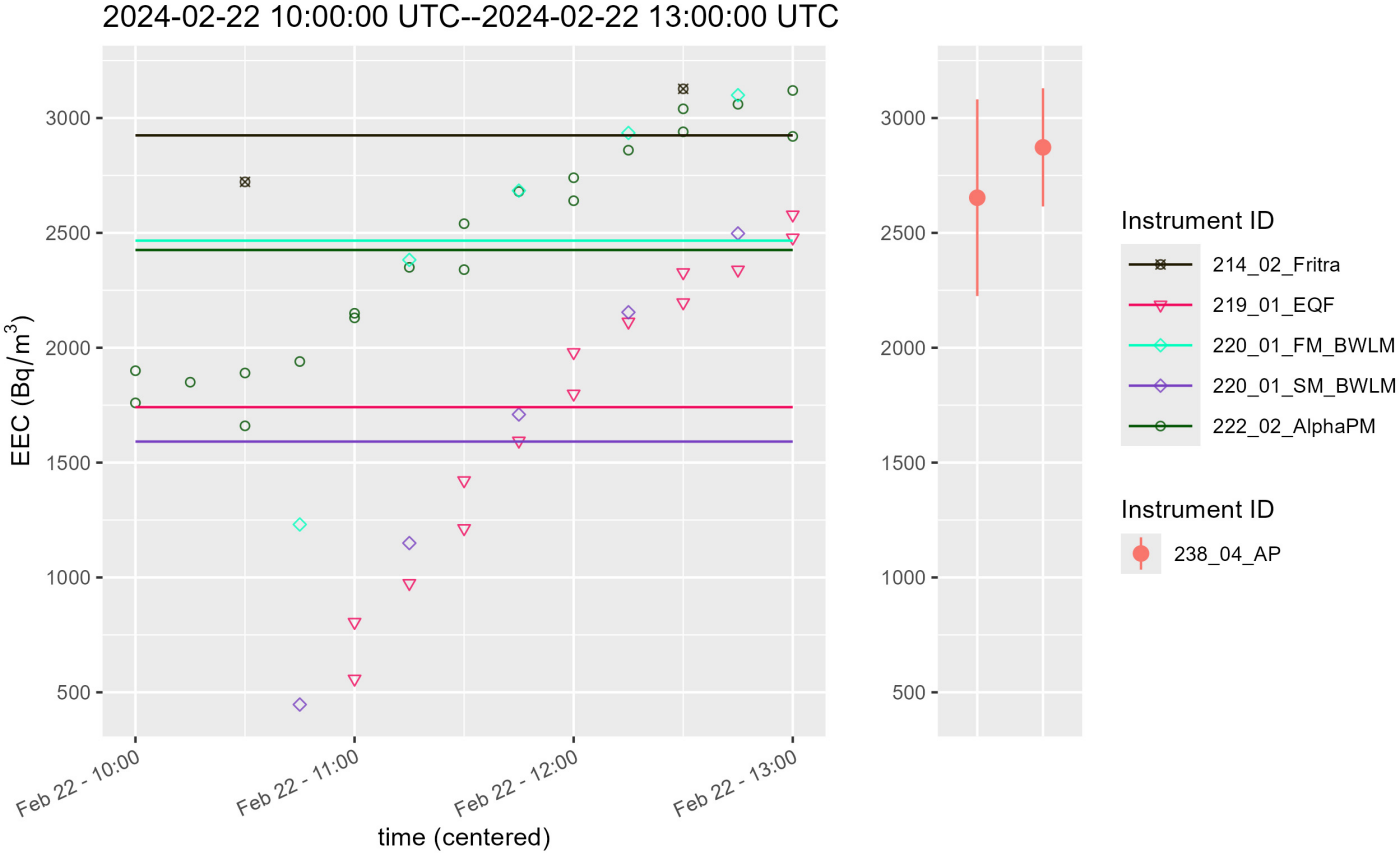
Comparison of EEC continuous **(left)** and integral measurement **(right)**, period III.

This comparison measurement provided a great opportunity to compare results from continuous monitors to integral ones, namely Alpha probes which are used for personal monitoring in active mines in Poland. The following graphs, Figures 9, 10, Supplementary Figure 4, provide time courses for EEC, the mean value of EEC from continuous monitors (left hand side of the graph) and result from integral measurement done by Alpha probe (right part of the graph). Complete set of data for this comparison are from 4 instruments—EQF 3220, AlphaPM, BWLM-PLUS-2S and FRITRA 4. The mean value from continuous monitors is illustrated by a line with the same color. Because the measurement periods were short, only 1–2 measurement results were available for FRITRA 4. The results from Alpha probes are presented with error bars provided by the instrument operator.

During period III, see Figure 10, the EQF 3220 had a drop out (measurements at the beginning of interval III are missing) and BWLM-PLUS-2S was starting after the change of mode, therefore a slow start of these two instruments can be seen (for BWLM-PLUS-2S only in slow mode). Hence the mean values of these instruments may be biased down.

**Figure 11:**
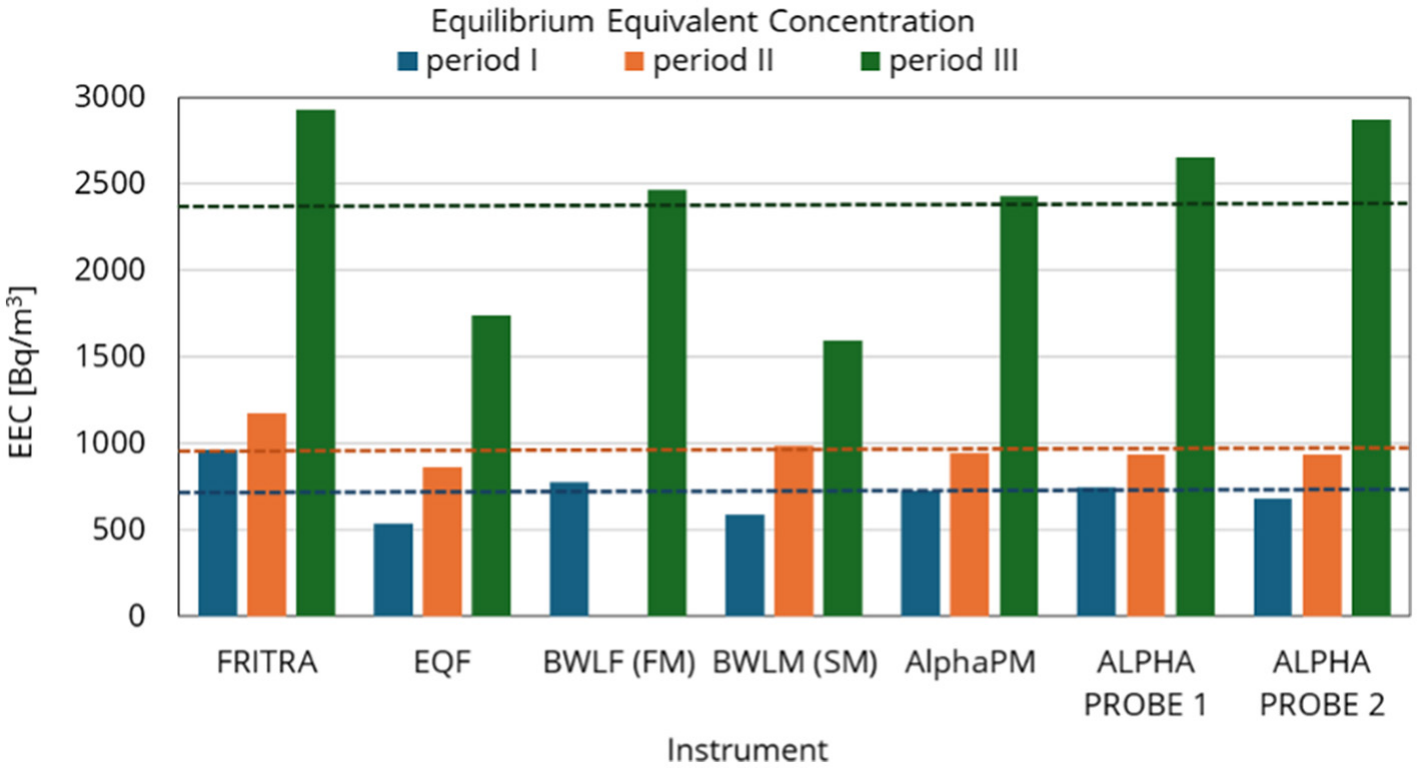
Comparison of EEC averages for periods I to III determined by continuous monitors and integral measurement; dashed line is the average value.

To summarize, there is a good agreement between the results, although the slow mode BWLM-PLUS-2S and EQF 3220 generally give lower values than the other measurements as could be seen from Figure 11.

Knowing the radon activity concentration and equivalent equilibrium radon concentration, it is possible to calculate the equilibrium factor ***F***. The Supplementary Figure 5 displays the time course of calculated values of *F*. As mentioned in previous sections 219-EQF 3220 had a drop out of 1 h and half on Feb-22 around noon (visible as a sharp drop in value). The 226-EQF 3220 started the third day due to prolonged stay at customs.

### 3.3 Unattached radon progeny and unattached fraction

**Figure 12:**
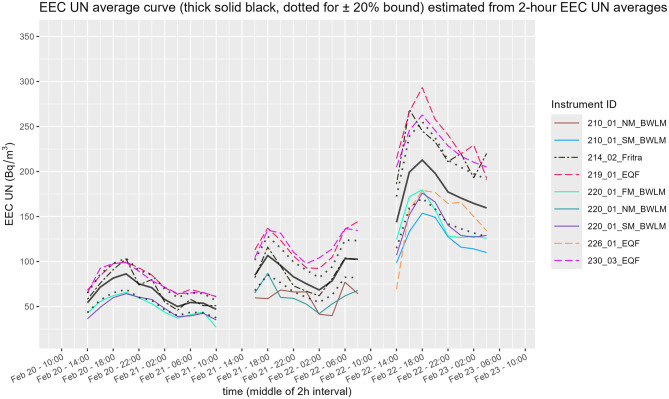
Time course of unattached radon progeny concentration of EEC_UN_ (SM, slow mode; NM, nuclide mode; FM, fast mode).

The results of the unattached radon progeny measurement EEC_UN_ are presented in Figure 12 for 6 instruments−3 EQF 3220, 2 BWLM-PLUS-2S and 1 FRITRA. Only two instruments measured the whole period of 3 days. BWLM-PLUS-2S instruments changed purposefully their mode twice (continuous and nuclide mode of measurement), the 219-EQF 3220 had a 1.5 h drop out (visible also in previous sections) and the 226-EQF 3220 started the measurement late. Because of the drop outs the three-day time period was divided into three sections:

- Feb 20 13:00–Feb 21 11:00
- Feb 21 15:00–Feb 22 9:00
- Feb 22 13:00–Feb 23 5:00.

To compare short EEC_UN_ time series from different instruments with varying sampling intervals, 2-h EEC_UN_ averages were used (as was done for Rn and EEC). From these 2-h averages, a mean curve with ±20 % bounds for each time section was calculated. The EEC_UN_ results appear more dispersed than the Rn or EEC measurements. Results from the 219-EQF 3220 and 230-EQF 3220 were more than 20 % above the mean curve. In contrast, both BWLM-PLUS-2S instruments were at the lower limit of the −20 % bound, while the 226-EQF 3220 fell only partially below it. The continuous modes of the BWLM-PLUS-2S instruments in the third time section showed a good agreement.

**Figure 13:**
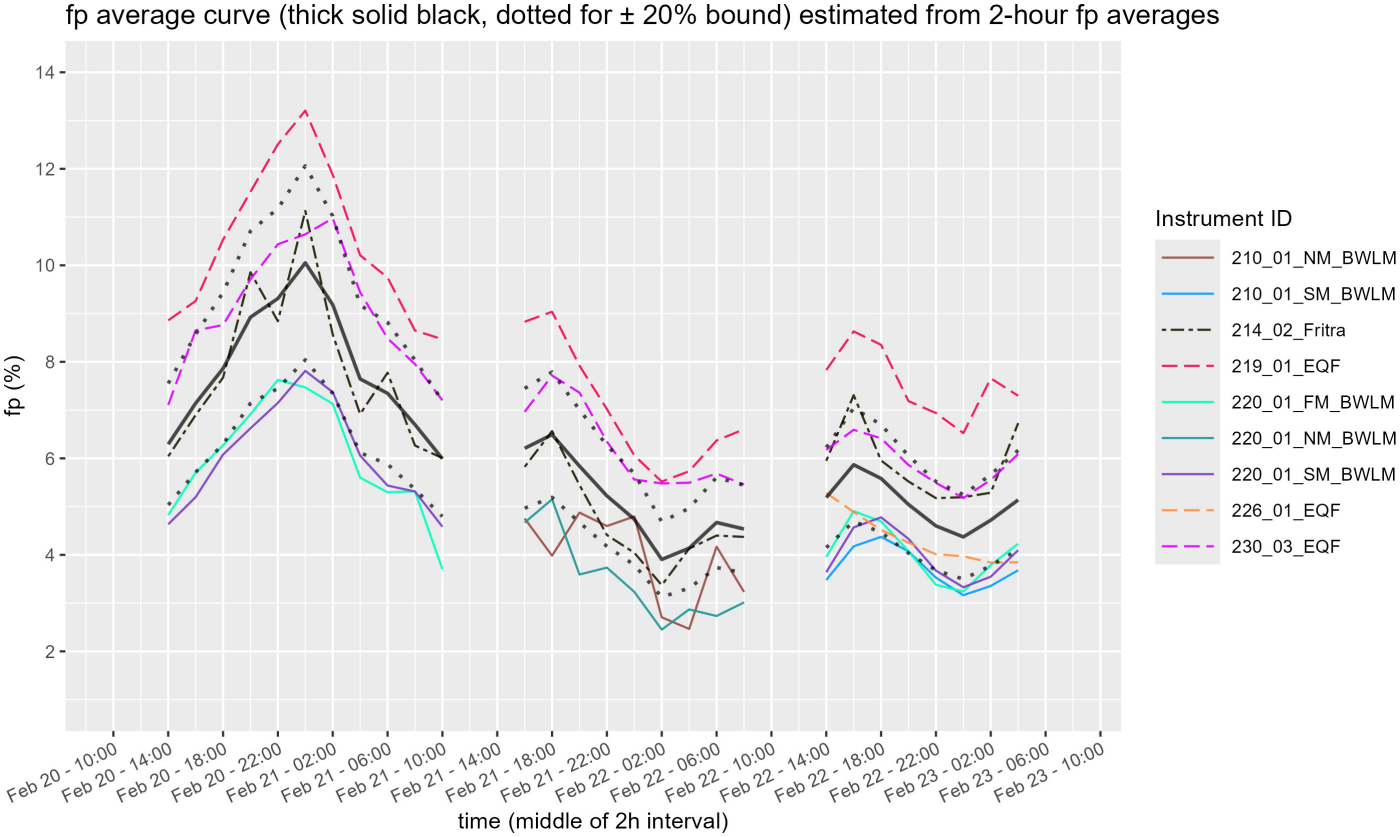
Time course of unattached fraction f_p_ (SM, slow mode; NM, nuclide mode; FM, fast mode).

Calculated values of unattached fraction *f*_*p*_ from total EEC and EEC_UN_ are presented in Figure 13.

### 3.4 Ambient conditions

**Figure 14:**
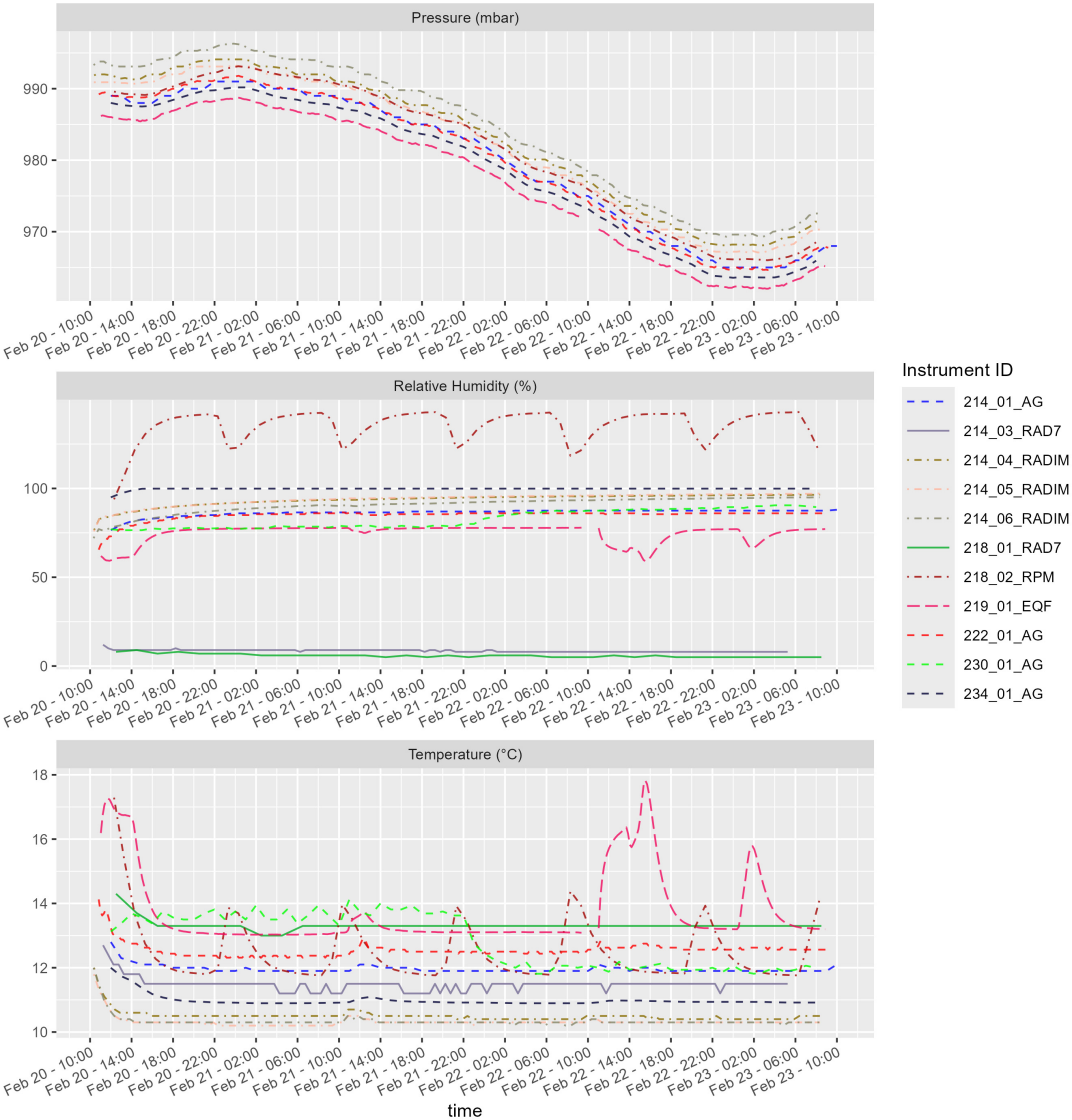
Results of measurement of additional parameters describing the ambient conditions.

Continuous monitors often provide measurements of ambient conditions, typically including temperature and relative humidity, and less frequently atmospheric pressure. These results are often overlooked, typically with the argument that the sensors are not calibrated. Results of monitoring of parameters of ambient conditions collected from continuous monitors during this comparison measurement are displayed in Figure 14. From the graph we can see peculiar results of relative humidity and temperature for at least 1 continuous monitor. The two lowest lines in the graph showing the relative humidity time course correspond to the RAD7 monitors, which operate with desiccant. Therefore, the measured values are low.

Time course of the particle concentration is shown in Supplementary Figure 6, where it can be seen that particle concentration varied from 1,600 to 5,200 particles/cm^3^, with an average of 3,000 particles/cm^3^. These values are typical for rather clean atmospheres, where few aerosols are generated, as is the case in tourist caves with little outside air intake through ventilation. Particle concentration measurements can be used to simulate the attachment of radon progeny to aerosols and estimate the unattached fraction, although this is beyond the scope of this paper.

## 4 Discussion

From the results of comparison measurement in field condition of underground workplace, namely Historical Silver mine in Tarnowskie Góry, we can see the following:

- Radon concentration varied from 1,000 to 5,000 Bq/m^3^.
- Comparison of results of measurement of radon gas activity concentration showed good agreement during this short measurement period (3 days only).
- For 4 identified stationary periods, all the average radon concentration provided by the participants from 15 continuous monitors fall within 20 % bound from the grand mean, with 3 exceptions—FRITRA 4 in P2, RADIM3A and EQF3220 in P4. Most of the results are within 5 % bound from the grand mean. FRITRA4 monitor was not originally intended for radon gas concentration measurement in variable conditions. Due to the measurement protocol, 10 min sampling each 2 h, the results obtained can be too rough for conditions with variable radon concentration.
- Equilibrium equivalent activity concentration (EEC) varied from 500 to 4,000 Bq/m^3^.
- Only 2 sufficiently long stationary periods were identified for the EEC results. The range of averages is wider compared to radon activity concentration measurement. All 10 instrument which provided results fall within the 20 % bound from the grand mean with 1 exception, RPM in P4. Due to time constraint, the RPM had not been calibrated in a radon calibration facility before the intercomparison but had only the manufacturer calibration performed after its repair.
- Comparison of short term (3–4 h) averages of EEC (PAEC) obtained from continuous monitors and integral TLD-based Alpha probes showed good agreement. Better results were provided for fast mode of BWLM-PLUS-2S monitor due to physical reasons. When evaluating the data, e.g. for dose calculation, the delay of the measurement results compared to reality have to be considered. The effective delay between exposure and reported values depends on the measurement mode: in fast mode, results are available after about 20–30 min, while in slow mode the response time constant is roughly 2 h. The slow mode provides lower statistical uncertainty, but its long delay makes it less suitable for situations where workers are present only for short periods. In such cases, fast mode or personal dosimeters should be used to ensure dose calculations reflect the actual temporal exposure pattern.
- Unattached fraction varied from about 5 % to 8 %.
- Equilibrium factor F varied from 0.6 to 0.8.
- Measured particle concentration varied from 1,600 to 5,200 particles/cm^3^, with an average of 3,000 particles/cm^3^.

The variability of radon and radon progeny concentration was quite high during the 3 days of inter-comparison measurement; however, the variability of concentration did not correspond to the opening hours or operating schedule of the ventilation system.

Already published data from the Historical Silver Mine in Tarnowskie Góry related to radon problem include both short-term (42) and long-term (29) measurements. Short-term measurement results conducted in March 2023 provide radon concentration as well as equilibrium factor F values, both show high variability between the individual measurement points. Radon concentrations ranged from 810 Bq/m^3^ to over 5,600 Bq/m^3^; equilibrium factor F varied from 0.15 to 0.94. For measurement point in the “Srebrna” chamber, values of 2,010 Bq/m^3^ for radon concentration and 0.67 for equilibrium factor F were reported. The results obtained within the inter-comparison are in good agreement with published short-term measurement results obtained for the particular location/measurement point.

Long-term results are available only for radon activity concentration (29). Long-term measurements were carried out by the use of CR-39 detectors from Radosys deployed at 30 measurement locations for 4 consecutive 3 months period (February–May, May–August, August–November, November–March). “Srebrna” chamber was measured at the inlet to and the outlet from the chamber. Results obtained from these two measurement points are in a good agreement—within a statistical error—for all 4 monitored periods. Average radon concentration obtained during summer and autumn period was almost double (1,440 Bq/m^3^) of the average radon concentration obtained during spring and winter (830 Bq/m^3^). On the other hand, closer look at the particular results reveals some variability—summer and autumn results ranged from 1,280 to 1,620 Bq/m^3^, spring and winter results ranged from 520 to 1,210 Bq/m^3^ (29).

High radon variability in underground workplaces is an often reported finding (43–47).

Although the quantities such as temperature, relative humidity or pressure are not of the main interest from the point of view of radiation protection of the worker and therefore the sensors are not the primary target of calibrations, they may provide additional and useful information for data processing and understanding. Moreover, all of the continuous monitors compensate for humidity and temperature, therefore their correct measurement results are reflected in radon activity concentration/equilibrium equivalent activity concentration.

## 5 Conclusions

A total of nine laboratories from seven European countries participated in the inter-comparison measurement conducted in the Historical Silver Mine Tarnowskie Góry, contributing sixteen continuous radon monitors, ten equilibrium activity monitors, and one TLD-based integrating system for PAEC measurement. Despite the short duration of the field campaign (three days), the comparison of radon activity concentration measurements showed strong consistency across most instruments. For four identified stationary periods, the majority of results from fifteen continuous monitors fell within a 20 % margin of the grand mean, with most of them within 5 %. Notable deviations were observed in three instruments. EEC measurements also demonstrated good agreement, with only one outlier among ten instruments. The comparison between continuous and integrating systems for PAEC yielded promising results, particularly with fast-response monitors like BWLM-PLUS-2S.

The study revealed significant variability in radon and progeny concentrations, ranging from 1,000 to 5,000 Bq/m3 for radon and 500 to 4,000 Bq/m3 for EEC, with equilibrium factors between 0.6 and 0.8 and unattached fractions from 5 % to 8 %. This variability did not correlate with the mine's operating hours or ventilation schedule, suggesting that other environmental or structural factors may influence aerosol dynamics.

Several factors can pose challenges to radon measurement instruments, or the measurement process itself. These include high humidity, airborne dust, limited or absent power supply, difficult accessibility, low temperatures, and insufficient availability of suitable instruments.

Currently, the measurement of radon progeny is approached more as a scientific discipline rather than a routine activity. However, given the EU BSS requirements for workplace monitoring, it can be expected that the number of measured workplaces with high radon concentrations will gradually increase. In many of them, due to workplace conditions, it will be necessary to determine the PAEC, or the concentration of the attached and unattached fractions of radon progeny. Given the already described variability of workplace conditions, there will likely be a shift toward continuous monitoring of progeny or the implementation of personal dosimetry.

Ensuring metrological traceability and regular (annual) in-house control of the instruments used is important to ensure quality and reliability of results. It should also be emphasized, that all currently available continuous monitors both for radon and for radon progeny do not allow to measure at above 90 or 95 % of relative humidity non-condensing, some do accept only even lower values. On the other hand, from the experience of the authors, the need to carry out continuous measurement at workplaces with very high relative humidity is high from both a radiation protection point of view as well as scientific interest. Therefore, it is advisable to test the instruments' response under simulated but controlled conditions, such as low temperature, high relative humidity, and rapid changes in radon concentration, as these are conditions that are at the edge of the instrument's operating conditions. This will avoid collection of misleading measurement results in the field.

## Data Availability

The raw data supporting the conclusions of this article will be made available by the authors, without undue reservation.
