# Supplementary material for: Intercomparison of radon and radon progeny concentration measurements performed in the Historic Silver Mine in Tarnowskie Góry, Poland
Source: Front Public Health. 2025 Oct 15;13:1681537. doi: 10.3389/fpubh.2025.1681537 (PMC12568597; doi:10.3389/fpubh.2025.1681537)
Supplement: Supplementary file 1 [file Image_1.pdf]

Intercomparison of radon and radon progeny concentration measurements performed in the Historic Silver Mine in Tarnowskie Góry, Poland

Katerina Navratilova Rovenska[1*], Miriam Slezakova[1], Caroline Vignaud[2], Pascale Blanchart[2], Katarzyna Wołoszczuk[3], Jostein Hoftuft[4], Agata Grygier[5], Krystian Skubacz[5], Arturo Vargas[6], Andrea Maiorana[7], Valeria Gruber[8], Joachim Gräser[8], Claudia Grossi[6, 9], Victòria Moreno[10], Lluís Font[10] and Karel Jílek[1]

[1] Statni ustav radiacni ochrany, v.v.i., Prague, Czech Republic,
[2] Direction de la recherche et de l'expertise en environnement, Autorité de Sûreté Nucléaire et de Radioprotection, Montrouge, France,
[3] Centralne Laboratorium Ochrony Radiologicznej, Warsaw, Poland,
[4] Direktoratet for strålevern og atomsikkerhet, Østerås, Norway,
[5] Główny Instytut Górnictwa-Państwowy Instytut Badawczy, Katowice, Poland,
[6] Institute of Energy Technologies, Universitat Politècnica de Catalunya, Barcelona, Spain,
[7] Centro Nazionale per la Protezione dalle Radiazioni e Fisica Computazionale, Istituto Superiore di Sanità, Rome, Italy,
[8] Österreichische Agentur für Gesundheit und Ernährungssicherheit (AGES), Abteilung Radon und Radioökologie, Linz, Austria,
[9] Physics Department, Universitat Politècnica de Catalunya, Barcelona, Spain,
[10] Departament de Física, Universitat Autònoma de Barcelona, Barcelona, (Spain)

*correspondence: katerina.rovenska@suro.cz

Supplementary Material

1 Supplementary Data

Due to space limitations, additional data and graphs are provided in the Supplementary Material.

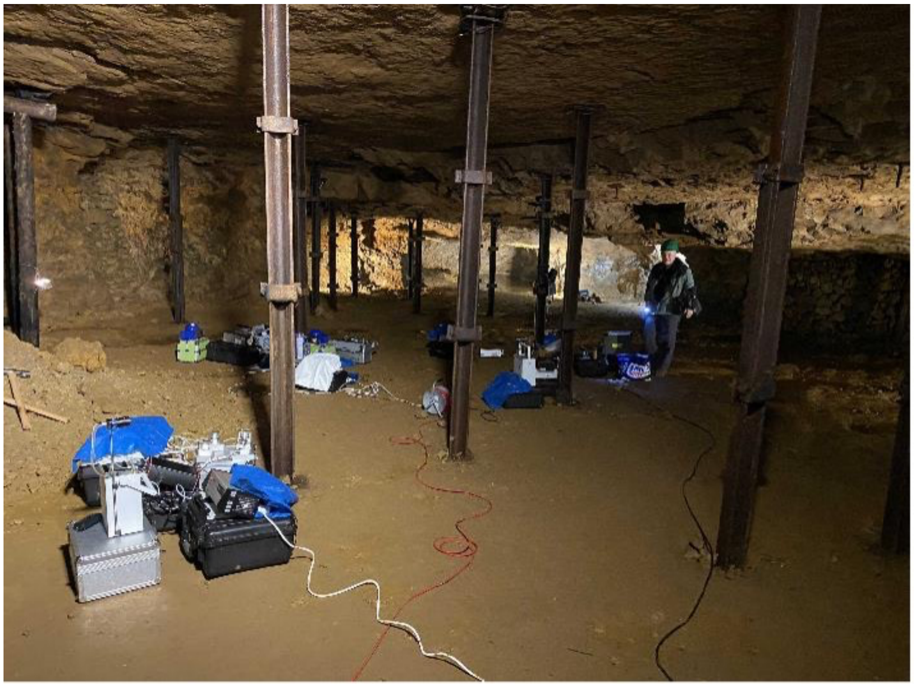

Supplementary Figure 1: Overview of the "Srebrna" chamber, with the monitors in place.

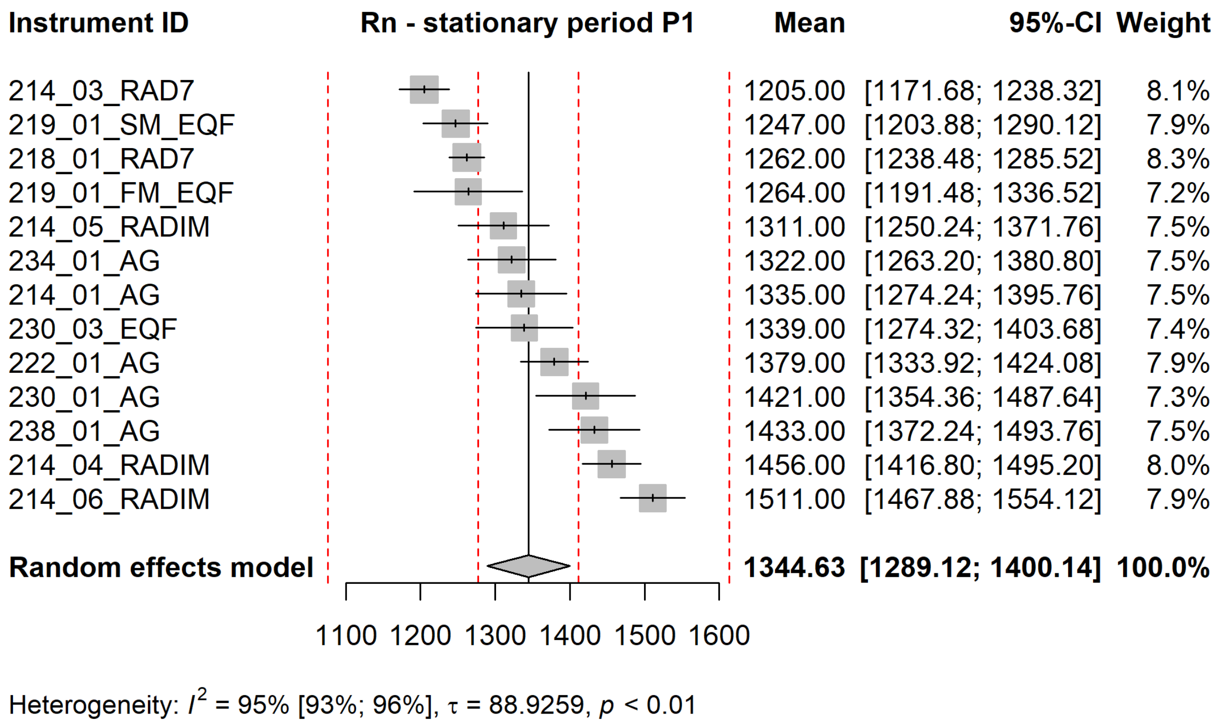

Supplementary Figure 2: Forest plot of stationary period P1, Rn measurement. The red dotted lines are ±5% and ±20% deviations from the grand mean.

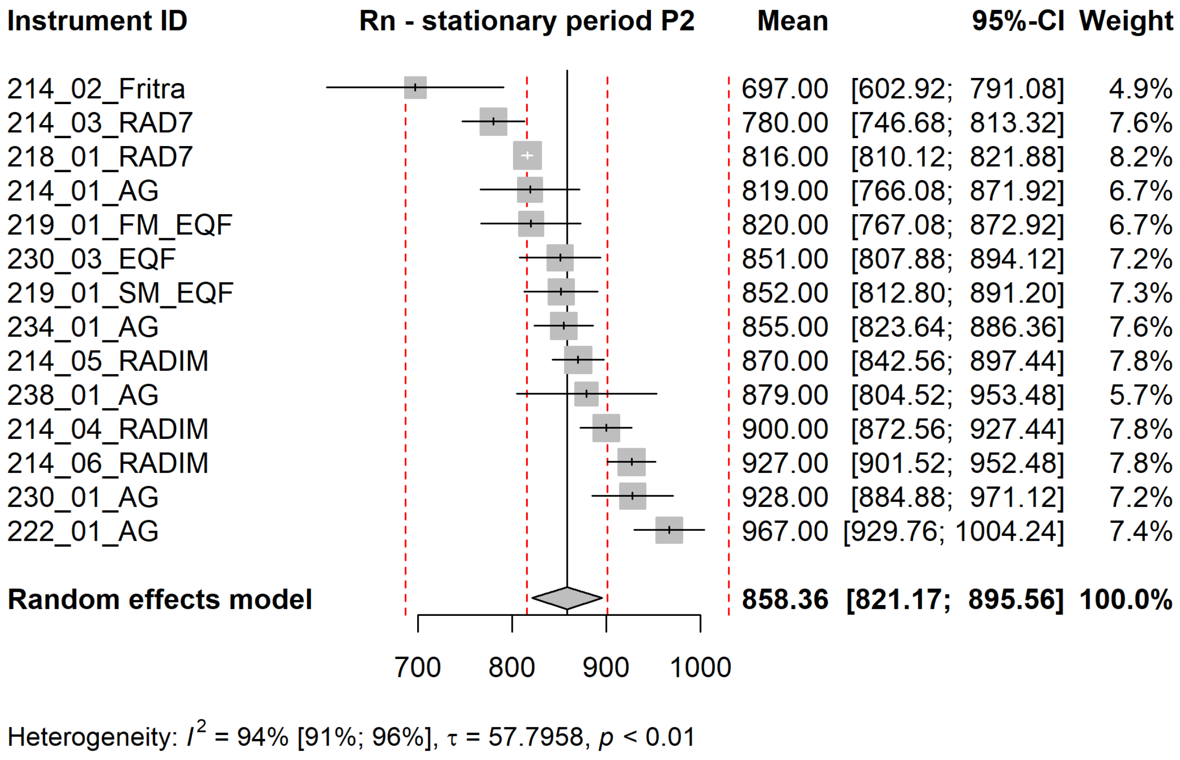

Supplementary Figure 3: Forest plot for stationary period P2, Rn measurement. The red dotted lines are ±5% and ±20% deviations from the grand mean.

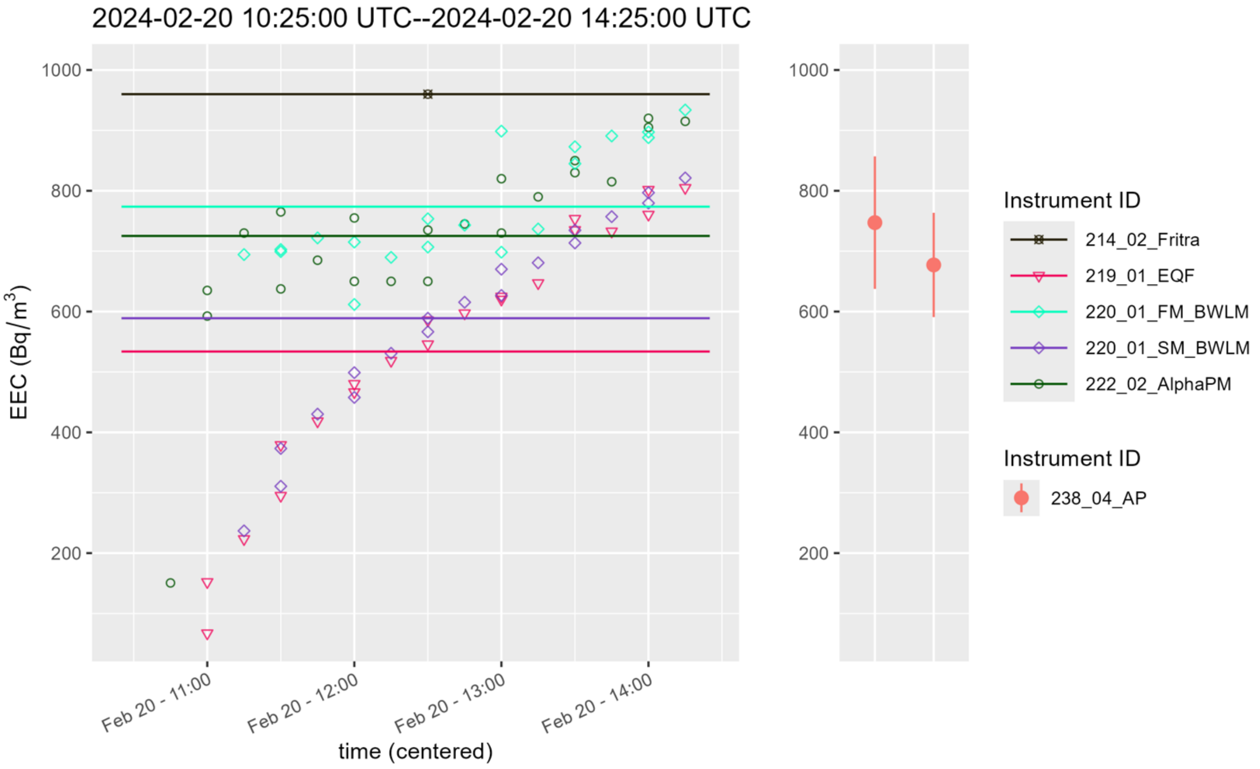

Supplementary Figure 4: Comparison of EEC continuous (left) and integral measurement (right), period I. Note that period I includes also the beginning of the comparison measurement, where all the instruments were starting. This could result in higher uncertainty, especially for EQF 3220 and BWLM-PLUS-2S (slow mode) instruments that have a slow start.

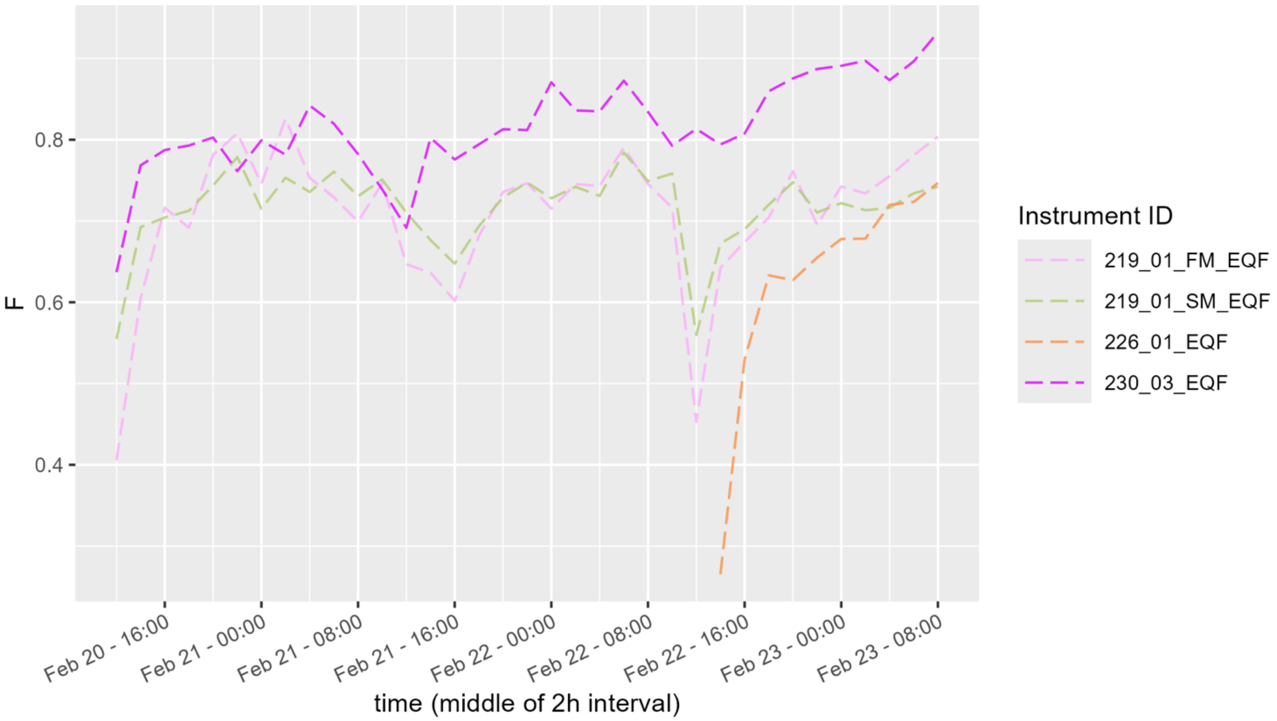

Supplementary Figure 5: Time course of calculated values of equilibrium factor F.

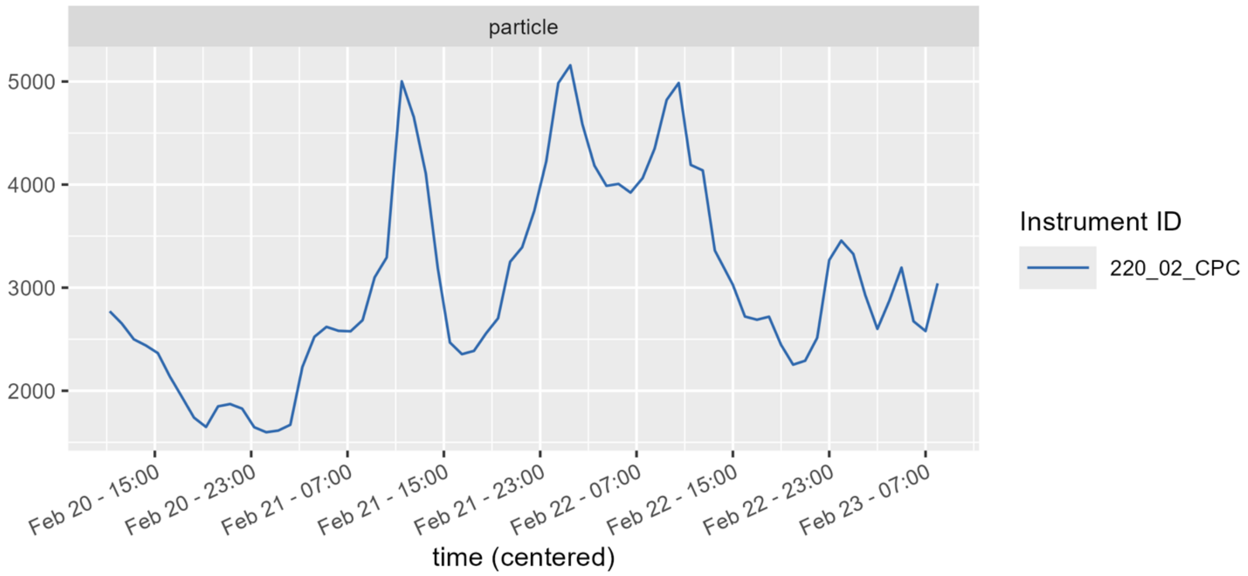

Supplementary Figure 6: Results of measurement of particle concentration particles/cm^3.
